# Elderly CD8^+^ T cells in the focus for immunotherapeutic approaches

**DOI:** 10.18632/aging.204747

**Published:** 2023-05-18

**Authors:** Annette Lis, Dorina Zöphel

**Affiliations:** 1 Biophysics, Center for Integrative Physiology and Molecular Medicine, School of Medicine, Saarland University, 66421 Homburg, Germany

**Keywords:** CD8 T cells ^+^, aging, cytotoxicity, immunotherapy

Immunosenescence is an inevitable phenomenon of the aging process and leads to increased susceptibility to severe infections and tumor diseases. At cancer diagnosis, the majority of patients are older than 65 years but this age group is often underrepresented in clinical trials of new immunotherapies. Due to comorbidities and immunosenescence, older patients are often considered ineligible, resulting in inadequate benefit/risk assessment of new treatment strategies [1, 2]. Modern immunotherapeutic approaches, such as adoptive T cell transfer, utilize patient-derived CD8^+^ T cells for targeted sensitization against tumor cells. Commonly reported defects of CD8^+^ T cells in old age question the success of T cell-based immunotherapies in elderly patients. However, due to the lack of single-cell based *in vitro* studies, the discrimination of functional defects caused by cell-intrinsic alterations or the suboptimal microenvironment and impaired antigen presentation in the aged host remains challenging. Nevertheless, elucidating intrinsically conserved capabilities may be crucial for selecting appropriate immunotherapies.

The diminished adaptive immune response in old age is often associated with reduced cytotoxicity of CD8^+^ T cells. However, surprisingly little is known about the maintenance of cytotoxic effector function throughout life.

Previous studies in aging mouse models confirm the impaired cytotoxic capacity of CD8^+^ T cells after bacterial or viral infection. In such infection models, the initiation of effector functions occurs *in vivo*, providing essential information about the influence of the aging environment on T cells’ immune response but giving little insight into the cellular cytotoxic capacity of the cells themselves.

So far, few studies allow an assessment of cell-intrinsic cytotoxic function with age showing significantly higher cytotoxicity of murine splenocytes against various tumor cell lines [3, 4]. However, the influence of CD8^+^ T cells´ cytotoxicity within the splenocyte population remains unclear. To close this important gap, we performed a comprehensive analysis of age-related functional changes in CD8^+^ T cells from elderly mice [5].

Time-resolved quantification of cytotoxicity for cell populations and on single-cell level revealed significantly faster lysis ability in the elderly, characterized by steep lysis kinetics immediately after target cell contact compared to CD8^+^ T cells from adult mice. Further analysis of the cytotoxic granule content showed significantly increased perforin and granzyme levels, turning the CD8^+^ T cells into very efficient killers. Noteworthy, this is not only true for the total CD8^+^ population. Both central (T_CM_) and effector memory (T_EM_) CD8^+^ T cells from elderly mice display a much more efficient cytotoxicity than respective subpopulations from the adults [5]. These results underline the assumption that the age-related inadequate elimination of pathogens and tumor cells *in vivo* is not due to intrinsic cell alterations in CD8^+^ T cell cytotoxicity. Consistent with the findings of Saxena et al., cell-intrinsic cytotoxic capacity increases with age. Thus, contrary to expectations, adoptive T-cell therapies may be particularly promising in elderly patients.

Currently, adoptive T-cell transfer is predominantly based on the total T cell population, and efficacy is highly influenced by individual subtype distribution. Although often hampered by quantitative limitations, numerous studies suggest the benefit of using defined T-cell populations to optimize current treatment strategies. Quantification of human T cell subpopulations` cytotoxicity on a single-cell level revealed higher cytotoxic efficiency of T_EM_ compared to T_CM _[6]. On the other hand, T_CM_ seem to supply better *in vivo* persistence and anti-tumor immunity after adoptive transfer [7, 8].

Surprisingly, we find that, despite immunosenescence, CD8^+^ T cell efficiency against cancer cells increases with age if similar CD8^+^ T cell numbers or individual cells are compared. The elevated perforin and granzyme levels turn the CD8^+^ T cells of elderly mice into ultimate killers. If these results also apply to CD8^+^ T cells from the elderly, this could open new approaches in immunotherapy, especially for elderly patients. On the other hand, it highlights the importance of distinguishing cell-intrinsic alterations from microenvironmental changes in elderly individuals.
